# Predictors of Mortality Following Aortic Valve Replacement in Aortic Stenosis Patients

**DOI:** 10.3390/pathophysiology29010010

**Published:** 2022-03-09

**Authors:** Vladimir Shvartz, Maria Sokolskaya, Andrey Petrosyan, Artak Ispiryan, Sergey Donakanyan, Leo Bockeria, Olga Bockeria

**Affiliations:** Department of Surgical Treatment for Interactive Pathology, Bakoulev Scientific Center for Cardiovascular Surgery, Rublevskoe Shosse, 135, 121552 Moscow, Russia; sokolskayam@mail.ru (M.S.); doctorapex@gmail.com (A.P.); ayispiryan@bakulev.ru (A.I.); sadonakanyan@bakulev.ru (S.D.); bockeria@bakulev.ru (L.B.); soleo2003@gmail.com (O.B.)

**Keywords:** aortic stenosis, predictors of adverse outcomes, aortic valve replacement

## Abstract

Background: Understanding of the risk factors for the development of adverse outcomes after aortic valve replacement is necessary to develop timely preventive measures and to improve the results of surgical treatment. Methods: We analyzed patients with aortic stenosis (*n* = 742) who underwent surgical treatment in the period 2014–2020. The average age was 63 (57;69) years—men 58%, women 42%. Results: The hospital mortality rate was 3% (22 patients). The following statistically significant threshold values (cut-off points) were obtained in the ROC analysis: aortic cross-clamp time > 93 min AUC (CI) 0.676 (0.640–0.710), *p* = 0.010; cardiopulmonary bypass time > 144 min AUC (CI) 0.809 (0.778–0.837), *p* < 0.0001, hemoglobin before op <120 g/L. AUC (CI) 0.762 (0.728–0.793), *p* < 0.0001, hematocrit before op <39% AUC (CI) 0.755 (0.721–0.786), *p* < 0.001, end-diastolic dimension index >2.39 AUC (CI) 0.647 (0.607–0.686), *p* = 0.014, end-systolic dimension index > 1.68 AUC (CI) 0.657 (0.617–0.695), *p* = 0.009. Statistically significant independent predictors of hospital mortality were identified: BMI > 30 kg/m^2^ (OR 2.84; CI 1.15–7.01), ischemic heart disease (OR 3.65; CI 1.01–13.2), diabetes (OR 3.88; CI 1.38–10.9), frequent ventricular ectopy before operation (OR 9.78; CI 1.91–50.2), mitral valve repair (OR 4.47; CI 1.76–11.3), tricuspid valve repair (OR 3.06; CI 1.09–8.58), 3 and more procedures (OR 4.44; CI 1.67–11.8). Conclusions: The hospital mortality rate was 3%. The main indicators associated with the risk of death were: diabetes, overweight (body mass index more than 30 kg/m^2^), frequent ventricular ectopy before surgery, hemoglobin level below 120 g/L, hematocrit level below 39%, longer cardiopulmonary bypass time and aortic cross-clamp time, additional mitral and tricuspid valve interventions.

## 1. Introduction

Aortic valve stenosis is among the most common pathologies of the heart valve apparatus, which is diagnosed in 2–5% of the adult population [1]. According to numerous studies, the defect incidence tends to increase with age. Consequently, aortic stenosis is diagnosed in 4–12% of cases in patients over 65 years old and in 20% in the group over 80 years old [1,2,3,4,5].

The choice of surgical intervention approach and surgery timing are based on a comprehensive decision based on examination results, surgery risk, presence of concomitant pathology and a patient’s fragility. The decision is made by the multidisciplinary team including a cardiologist, a cardiac surgeon, an X-ray endovascular surgeon, and a diagnostician [1,6,7,8,9,10,11,12,13]. Aortic valve replacement is recommended for most patients with severe aortic stenosis, whereas conservative treatment neither improves survival, nor slows down the progression of this defect [4,6,7]. Surgical aortic valve replacement (SAVR) of the affected valve represents standard treatment in the low risk group of patients (<75 years and STS-PROM/EuroSCORE II < 4%) or in patients who are operable and unsuitable for transcatheter aortic valve implantation (TAVI). In patients with high and extremely high risk of surgical treatment (STS-PROM/EuroScore II > 8%) and in older patients (>75 years) or those unsuitable for surgery, transcatheter valve replacement is recommended. Additionally, the choice of SAVR or TAVI depends on individual clinical, anatomical and procedural characteristics, and the values and expectations of the informed patient. Patients whose life expectancy is under a year are considered inoperable [4,6,8,10,11,13].

In a contemporary cardiac surgery, an overall 30-day mortality rate with isolated aortic valve replacement in patients with normal contractility of the left ventricular myocardium is 1–4%; however, the presence of concomitant cardiac or comorbid pathology significantly increases the risk of unfavorable outcomes. Accordingly, the combination of aortic valve replacement with coronary artery bypass grafting (CABG) results in a mortality increase of up to 4.5% [10].

Because elderly patients with multiple comorbid pathologies are increasingly registered in actual recent clinical practice, it is important to determine the role of concomitant diseases as risk factors for unfavorable outcomes of the surgery [6,8]. The study objective was to identify the predictors of mortality in the early postoperative period in aortic stenosis patients after the aortic valve replacement.

## 2. Materials and Methods

### 2.1. Study Population

A retrospective cohort follow-up study of the patients who underwent surgical interventions for aortic valve pathology was conducted. The general database comprised the surgical aortic valve replacement patients with mechanical and biological prostheses, with transcatheter valve replacement, with reconstructive valve-sparing surgeries on the aortic valve, etc. The aortic valve pathologies were caused by congenital anomaly (bicuspid aortic valve), defects of rheumatic etiology, degenerative valve damage, infective endocarditis, etc. Hemodynamically, these were the patients with predominant stenosis of aortic valve, aortic regurgitation, or combined lesion of the aortic valve.

This article includes an analysis of the hospital stay in aortic stenosis patients over the period 2014–2020, who underwent aortic valve replacement with a mechanical prosthesis.

Furthermore, the patients were selected from the same structural unit, specifically the department of surgical treatment of interactive pathology. This choice was justified by the fact that all these patients underwent surgery using the same technique (with retrograde cardioplegia).

### 2.2. Data Collection

The standard examination before the surgery included the collection of clinical and anamnestic data, as well as laboratory and instrumental studies. The concomitant comorbid pathology was confirmed with the involvement of specialists in this field (for example, an endocrinologist in case of diabetes mellitus, a pulmonologist with chronic obstructive pulmonary disease, etc.). Ischemic heart disease was confirmed, or ruled out, in all patients over 40 years old, using selective coronary angiography.

Transthoracic echocardiography was performed in all patients before the surgery. At the early stages after surgery, solely transthoracic echocardiography was used on a daily basis to evaluate hemodynamics, the presence of pericardial effusion, to assess the contractile function of the myocardium, etc. Laboratory diagnostics included assessment of blood parameters both before the surgery and daily after the surgery for 5 days, and additionally on the day of discharge from the clinic (usually, day 7 or 8 after the surgery). Electrocardiography (ECG) monitoring was performed at the clinic on the day 1 after the surgery using bedside monitors, followed by long-term Holter ECG monitoring until the discharge from the hospital.

Intraoperative data, the resuscitation, along with intensive care unit and early postoperative data were collected from the general electronic database of the “MedWork” clinic in compliance with all legal principles.

This study protocol conforms to the ethical guidelines of the 1975 Declaration of Helsinki in line with the Ethical Guidelines for Epidemiological Research by the Russian government. This study was approved by the Local Ethics Committee of Bakulev Center for Cardiovascular Surgery of the Ministry of Health of the Russian Federation. Written informed consent was obtained from each patient before each surgery by the physicians.

### 2.3. Definitions

The definition of cardiac rhythm disorder (CRD) combines the arrhythmias diagnosed via a 12-channel ECG or Holter ECG monitoring, specifically atrial fibrillation, auricular fluttering, ventricular tachycardia, frequent ventricular extrasystole (FVE) grades II–V sensu Lown-Wolf, tachybradycardia syndrome, and conduction disorders (e.g., atrioventricular blockade degree II–III). We considered FVE and atrioventricular blockade as separate risk factors in uni- and multivariate logistic regression. Myocardial infarction (MI) was primarily diagnosed based on a patient’s medical records and ECG-confirmed cicatricial changes in the myocardium. The history of MI/ACVA (acute cerebrovascular accident) was established based on anamnestic evidence provided by the patient, as well as on the results of CT/MRI of the brain. The patients were included in the group of chronic kidney disease (CKD) prior to the surgery based on estimated glomerular filtration rate (eGFR); those with eGFR of 59 mL/min/1.73 m^2^ or lower were classified as stage 3 or higher (stages 3–5). eGFR was calculated using the Cockcroft–Gault and MDRD (modification of diet in renal disease) formulae. The maze procedure is cryoablation and radiofrequency ablation of the left atrium in combination with correction of valvular heart disease. It includes cryoisolation of areas where reentry circles may occur (left atrial appendage, the pulmonary vein ostia, vena cava ostia, and coronary sinus area) in combination with plastic surgery—else, if it is impossible to preserve the native valve, with atrioventricular valve replacement.

### 2.4. Endpoint

The primary endpoint was represented by the hospital mortality. Secondary endpoints have not been studied.

### 2.5. Surgery

Aortic valve replacement with a mechanical prosthesis was performed on a scheduled basis; no emergency interventions were performed. Patients underwent standard sternotomy and conventional open chest cardiopulmonary bypass (CPB), moderate hypothermia, retrograde pharmacological and hypothermic cardioplegia. A median sternal incision was used to approach the heart. In cases of a combination of aortic valve replacement with CABG, the first stage was the collection of conduits in the planned amount, with subsequent aortic valve replacement. Then, after restoring the integrity of aorta and right atrium, a patient’s body was warmed up to 36.6 °C, and the cardiac activity was reestablished. Next, myocardial revascularization was performed on the beating heart. Bypass grafting of the target coronary arteries was carried out by imposing a distal anastomosis, followed by proximal anastomoses formed on the parietally deflated aorta. The quality of formed anastomoses was assessed using intraoperative bypass angiography, which enabled identification and elimination of their defects in a timely manner.

Various techniques were employed to correct the mitral valve, including sutural annuloplasty of the valve, or multicomponent valve reconstruction. In cases of unsuccessful grafting, assessed by intraoperative transesophageal echocardiography, valve replacement was performed. Tricuspid valve repair was conducted using De Vega technique. The maze procedure was used to correct atrial fibrillation [14].

### 2.6. Statistical Analysis

Our database comprised both quantitative and categorical variables. All quantitative data were examined for normality of distribution using the Shapiro–Wilk test. The data were not normally distributed. We used the nonparametric statistical methods. Continuous variables were analyzed using the Mann–Whitney U test. Fisher’s exact test or the χ2 test were used for categorical variables, whenever appropriate. The association between the hospital mortality and any comorbidities was evaluated using univariate logistic regression analyses and presented by odds ratio (OR), 95% confidence interval (CI), and *p*-value. When conducting the multivariate logistic regression analyses, the intercorrelated parameters were excluded, and the most significant parameters from the univariate logistic regression model were included in the model. To identify effective diagnostic cutoff points of continuous indicators under study, we used ROC analysis, by plotting the curve and evaluating the AUC (area under the curve). The data were presented as median and interquartile range Me (Q1; Q3) when describing quantitative data, and absolute number and proportion *n* (%) when describing categorical variables. The software packages STATISTICA 10 (Statsoft, Tulsa, OK, USA), Microsoft Office Excel, and MedCalc (MedCalc Software Ltd., Oostende, Belgium) were used.

## 3. Results

### 3.1. Characterization of Patients

The study included 742 patients: 429 men (58%) and 313 women (42%) 18–85 years old, with the median age of 63 (57; 69) years, and 65% of patients over the age of 60 years. Hospital mortality was 3% (i.e., 22 patients died). The main causes of death were: acute heart failure (*n* = 7), pneumonia (*n* = 5), bleeding (*n* = 4), cardiac arrhythmias (*n* = 3), acute renal failure (*n* = 1), complicated re-operation (*n* = 2).

Table 1 presents the clinical data on the patients. Instrumental and laboratory data, as well as data on medicamentous therapy of patients, are demonstrated in Table 2.

Table 1 implies that the incidence of comorbidities in the group of patients with lethal outcome was significantly higher. The comorbidities included diabetes mellitus (38% vs. 13%), coronary heart disease (79% vs. 50%), the history of myocardial infarction (21% vs. 6%), cardiac arrhythmias (41% vs. 23%), and diagnosed mitral valve defect (32% vs. 9.5%). In addition, patients were statistically significantly different in terms of their body mass index (BMI) values. Hence, in the group of patients with lethal outcome, BMI was 31 (28; 34) versus 28.4 (25; 32) in the other group. There were no statistically significant differences in medicamentous therapy prior to the surgery.

When comparing the groups according to the severity of chronic heart failure, no statistically significant differences were obtained (*p* = 0.849). Apparently, this was due to the fact that about 70% of patients in both groups were already in NYHA class III-IV. It is also known that surgery should be considered in asymptomatic patients and in patients with aortic stenosis at earlier stages in order to reduce the risk of surgical intervention. The use of echocardiography capabilities is an effective strategy in this cohort of patients [15,16].

Statistically significant differences were obtained for the end-diastolic dimension index and end-systolic dimension index: both were greater in the group of patients with a lethal outcome. Baseline hemoglobin and hematocrit levels were lower in the lethal outcome group.

The comparison of intraoperative and postoperative parameters reveals that both the time of CPB and the time of aortic cross-clamping were longer in the lethal outcome group (Table 3). Considering a complicated postoperative period in patients with lethal outcome, they stayed longer in the hospital, including the resuscitation department and intensive care unit.

### 3.2. ROC Curve Analysis

To identify effective cutoff points of the studied continuous indicators associated with lethal outcome, the ROC analysis was performed. The parameters of BMI higher than 30 U, the indices of end-diastolic dimension higher than 2.4 cm/m^2^ and end-systolic dimension higher than 1.7 cm/m^2^, the CPB duration longer than 144 min, aortic cross-clamping longer than 93 min, along with the levels of hemoglobin of 120 g/L or lower and hematocrit of 39% or lower, were statistically significant (Table 4). We used the binary division of continuous data into categorical data when calculating univariate and multivariate logistic regression models.

### 3.3. Uni- and Multivariate Logistic Regression Analysis

Univariate analysis yielded statistically significant independent predictors of lethal outcome: coronary heart disease, diabetes mellitus, FVE, surgical intervention on the mitral valve, more than three simultaneous interventions, etc. (Table 5). In multivariate logistic regression analysis, it was revealed that the most powerful predictive factors were FVE (*p* = 0.013) and CPB duration over 144 min (*p* = 0.028) (Table 6).

## 4. Discussion

At present, the aortic valve replacement is among the most frequently performed and safest cardiac surgeries. However, for older patients, those with comorbid pathology, combined lesions of several heart valves, and in case of multifocal atherosclerosis, the risk of lethal outcome of this surgery remains quite high [5,17]. To improve the results of surgical treatment in this category of patients, when determining the approach and scope of the forthcoming surgery and making a decision on its timing and necessity, a personalized approach with participation of a multidisciplinary team, taking into account all existing risk factors, is required [18,19,20,21].

According to the published sources, the lethal outcome rate in elderly patients is 5–10% [5]. In our study, hospital mortality was 3%, which is comparable to the global data. In our study, additional surgical intervention on the mitral valve was a statistically significant independent predictor of a lethal outcome, because the scope of the surgery increased, leading to longer times of CPB and aortic cross-clamping. Ischemic heart disease was a risk factor for mortality in the perioperative period; however, myocardial infarction in the anamnesis and combined surgery with CABG were not significant factors.

CPB is a unique innovation of the 20th century and an integral part of a cardiac surgery. However, despite the improvement of devices and techniques, it still remains a nonphysiological procedure causing disorders in the homeostasis of the entire organism. CPB causes the activation of systemic inflammation, mechanical trauma to the blood cells, hyperoxia, hemodilution, hypothermia and nonpulsatile blood flow. During CPB, when blood comes into contact with the apparatus surfaces, inflammatory reactions are activated, endothelial dysfunction is induced, and capillary permeability increases [22]. Correspondingly, longer times of CPB and aortic cross-clamping increase the risk of adverse events in the perioperative period. In our study, CPB time of over 144 min and aortic cross-clamping time of over 93 min were statistically significantly associated with lethal outcome.

Although FVEs are considered relatively benign, they have been associated in some studies with an increased risk of mortality from any cause, including cardiovascular diseases, both in patients with existing cardiovascular disease and in people without structural heart diseases. The main pathogenetic mechanism of increased mortality with FVE is triggering life-threatening rhythm disorders, primarily ventricular tachycardia [23,24,25,26].

Overweight status was also identified as an independent predictor of a lethal outcome. The publications present data on impaired breathing mechanics, reduction in a lung volume, deterioration of bronchial patency and ventilation-perfusion relations in obese patients. In addition, this group of patients is more often susceptible to infectious complications, such as pneumonia, wound and catheter-associated infections, sepsis, and renal failure [27].

When preparing patients for planned cardiac surgery, hematological parameters are no less important in terms of the surgery prognosis and outcome [25]. Anemia is well known as a modifiable risk factor for the development and progression of cardiovascular events [19,28,29]. A decrease in hemoglobin levels leads to tissue hypoxia, which results in irreversible changes in the entire body. With hypoxia, a patient undergoes vascular remodeling, proliferation of smooth muscle cells increases, and production of proinflammatory cytokines increases as well. A number of authors suggest that long-term anemia leads to remodeling of cardiac chambers, since with a decrease in oxygen content in the coronary blood flow, oxygen demand in the myocardium increases, which requires a greater cardiac output to compensate and maintain its function. If a patient has significant stenosis of the coronary arteries, then the anemia tolerance is even worse. Assessment of the hematocrit index prior to the surgery is an important routine. Excessive hemodilution leads to a decrease in the concentration of blood coagulation factors, and can also cause a decrease in oncotic pressure and interstitial edema, thereby increasing the risk of postoperative complications [22,30,31]. In our study, the baseline values of hemoglobin and hematocrit were associated with the risk of mortality. Hemoglobin levels below 120 g/L and hematocrit levels below 39% were independent predictors of a lethal outcome.

Chronic kidney disease is a known independent risk factor for cardiovascular events and a predictor of a lethal outcome after the open-heart surgery; it increases the incidence of postoperative atrial fibrillation and deteriorates the prognosis in diabetic patients [32]. In our study, the functional state of the kidneys was assessed, taking into account the indicators of creatinine level and eGFR, calculated by the Cockcroft–Gault formula and MDRD, taking into account age, gender, and body surface area. When comparing preoperative indicators, no statistically significant differences were detected between the groups. However, when analyzing these indicators on day 3 after the surgery, they differed significantly, eGFR was reduced, and creatinine level was increased in patients with poor outcomes. One of the criteria for a reduction in renal function is an indicator of the glomerular filtration level, and the eGFR in the range of 60–89 mL/min/1.73 m^2^ is regarded as its initial or insignificant decrease. In this case, the presence of markers of a renal damage is required to establish CKD. In the absence of any markers of renal damage, the diagnosis CKD can be made if the eGFR is below 60–89 mL/min/1.73 m^2^. Since nephropathy is an independent risk factor for the development of postoperative complications, the necessity of a more attentive attitude to this indicator and a decision on the issue of nephroprotection is quite obvious. An important component of nephroprotective therapy involves non-medicamentous treatment methods; attention should be paid to the use of a diet in this group of patients. Additionally, caution should be exercised when using such nephrotoxic drugs as aminoglycosides, vancomycin, nonsteroidal anti-inflammatory drugs, angiotensin-converting enzyme inhibitors, and angiotensin receptor antagonists, which are often used in the management of patients after the cardiac surgery [32,33,34,35,36].

Univariate analysis yielded statistically significant independent predictors of lethal outcome: coronary heart disease, diabetes mellitus, FVE, surgical intervention on the mitral valve, more than three simultaneous interventions, etc. (Table 5). In addition, statistically significant differences were obtained for the end-diastolic dimension index (LVEDD/BSA cm/m^2^ > 2.39) and end-systolic dimension index (LVESD/BSA cm/m^2^ > 1.68): both were greater in the group of patients with a lethal outcome (OR 5.01; CI 1.07–23.4 and OR 4.81; CI 1.03–22.5), respectively. Assessing the remodeling patterns of LV is an important step for the evaluation of patients with aortic stenosis, because it impacts on prognosis [37].

The use of multivariate logistic regression analysis often leads to the fact that more powerful statistical factors supersede those slightly less powerful (Table 6). Additionally, the parameters that are correlated with each other cannot be added to the model. In our case, for example, initially significant end-systolic dimension and end-diastolic dimension indices strongly correlate with each other. As a result, in the multivariate model in our cohort, the most powerful factors (with regression coefficients of about 2.5) were FVE and CPB time of over 144 min.

### Limitations of the Study

This analysis had some drawbacks related to the retrospective approach.

Firstly, despite the fact that all data were collected from the general electronic database of our clinic “Medwork”, with the standard mandatory data entry, the retrospective analysis does not exclude partial data loss.

Secondly, in order to minimize the impact of the surgical intervention technique in this study, we included patients from one department and the surgical procedures were performed using the same technique (the retrograde cardioplegia); nevertheless, this is an analysis of the work of several surgeons, and not an experience of one surgeon.

Thirdly, drug therapy in the early postoperative period was analyzed in detail, although guidelines were not strictly adhered to in every case and the therapy was individualized, if needed.

## 5. Conclusions

In our study, the hospital mortality after aortic valve replacement was 3%. The presence of concomitant diabetes mellitus in patients, overweight status (BMI over 30 kg/m^2^), FVE, increased systolic and diastolic dimensions of the left ventricle, as well as the levels of hemoglobin below 120 g/L and hematocrit below 39%, increased the risk of hospital mortality. Long periods of CPB time (over 144 min) and aortic cross-clamping time (over 93 min), and also greater complexity of a cardiac surgery, were also the risk factors. Minimizing the above predictors and using the concept of multidisciplinary team discussion of the patient would reduce the risks of poor outcome.

## Figures and Tables

**Table 1:** Clinical parameters of patients.

Parameters	All	Mortality	No Mortality	*p*
N	742	22	720	
Age, years	63(57; 69)	66 (62; 70)	63 (56; 69)	0.131
Male gender, %	58	45	58	0.225
BMI, kg/m^2^	28(25; 32)	31 (28; 34)	28.4 (25; 32)	0.025
Weight, kg	80 (70; 89)	78 (72; 92)	80 (70; 89)	0.620
BSA, m^2^	1.9 (1.85; 2.01)	1.9 (1.83; 2.04)	1.9 (1.86; 2.01)	0.125
Hypertension, *n* (%)	93	93	93	0.987
Smoking, *n* (%)	12	0	12	0.427
IHD, *n* (%)	51	79	50	0.035
Prior AMI, *n* (%)	7	21	6	0.022
Stroke, *n* (%)	4	0	4	0.409
Diabetes, *n* (%)	14	38	13	0.006
COPD, *n* (%)	6	7	6	0.803
CKD, *n* (%)	11.7	22.7	11	0.104
CHF NYHA class III-IV, %	71.5	69	71.6	0.849
ASD, *n* (%)	<1	7	<1	0.004
VSD, *n* (%)	<1	0	<1	0.871
MV disease, %	10	32	9.5	<0.001
TV disease, %	9.2	22.7	8.8	0.025
Arrhythmia	23	41	23	0.049
AF, %	13	18	12	0.419
Atrial flutter, %	2	0	2	0.588
FVE, %	2	14	2	<0.001
VT, %	<1	0	<1	0.844
Sick sinus syndrome, %	<1	0	<1	0.746
AV block, %	3	14	3	0.014
Sinus rhythm, %	87	93	87	0.692
Cerebrovascular disease, %	28	50	28	0.074
Peripheral vascular disease, %	22	36	22	0.223
Concomitant oncological disease, %	9	7	9	0.799

BMI—body mass index; BSA—body surface area; IHD—ischemic heart disease; AMI—acute myocardial infarction; COPD—chronic obstructive pulmonary disease; CKD—chronic kidney disease; CHF NYHA—chronic heart failure New York Heart Association classification; ASD—atrial septal disease; VSD—ventricular septal disease; MV—mitral valve; TV—tricuspid valve; AF—atrial fibrillation; FVE—frequent ventricular ectopy; VT—ventricular tachycardia; AV block—atrioventricular block.

**Table 2:** Instrumental and laboratory characteristics of patients and drug therapy before surgery.

Parameters	All	Mortality	No Mortality	*p*
N	742	22	720	
Echocardiographic parameters				
LVEF, %	64 (58; 67)	63 (58; 67)	64 (58; 67)	0.974
LVEDD/BSA, cm/m^2^	2.6 (2.3; 2.9)	2.99 (2.72; 3.77)	2.6 (2.3; 2.9)	0.001
LVESD/BSA, cm/m^2^	1.68(1.49; 1.93)	1.98 (1.75; 2.12)	1.67(1.49; 1.91)	0.008
LVEDV/BSA, mL/m^2^	61.5 (49; 78)	67 (59; 116)	61.4 (50; 78)	0.103
LVESV/BSA, mL/m^2^	22 (17; 30)	26 (22; 31)	22 (17; 30)	0.152
Peak gradient, mm Hg	96 (80; 112)	95 (86; 105)	96 (80; 112)	0.912
Peak velocity, m/s	3.5 (2.8; 4.2)	3.7 (2.9; 4.2)	3.5 (2.8; 4.2)	0.451
Mean gradient, mm Hg	55 (44; 66)	58 (45; 62)	54 (44; 67)	0.944
Fibrous ring of the aortic valve, mm	23 (22; 25)	22 (21; 25)	23 (22; 25)	0.174
Bicuspid aortic valve, %	14.8	9	15	0.201
Moderate AR, %	21	36	21	0.401
Severe AR, %	5.9	9	5.8	0.733
EOA, cm^2^	0.7 (0.55; 0.8)	0.65 (0.6; 0.8)	0.7 (0.55; 0.8)	0.233
LA volume, mL^3^	109 (90; 140)	132 (102; 145)	109 (90; 140)	0.542
Laboratory parameters				
Hemoglobin level, g/L	136 (126; 146)	118 (111; 134)	137 (127; 146)	<0.001
Hematocrit, %	41 (38; 44)	36.5 (35; 39)	41 (38; 44)	<0.001
WBC, 10^9^/L	7.2 (6; 8.6)	7.1 (6.6; 8.8)	7.2 (6; 8.6)	0.539
Neutrophils, 10^9^/L	4.6 (3.6; 5.6)	4.8 (3.7; 5.5)	4.6 (3.6; 5.6)	0.549
Neutrophils, %	60 (53; 65)	67 (58; 71)	59 (53; 65)	0.032
glucose, mmoL/L	5.3 (4.9; 5.9)	5.7 (5; 7,4)	5.3 (4.9; 5.9)	0.059
Fibrinogen, g/L	4.1 (3.7; 4.7)	4.7 (4.3; 4.9)	4.05 (3.6; 4.6)	0.064
Creatinine, mkmoL/L	82 (72; 96)	87 (69; 108)	82 (72; 96)	0.481
eGFR, mL/min	87 (69; 103)	75 (62; 100)	87 (69; 103)	0.129
eGFR, mL/min per 1,73 m^2^ (MDRD)	78(66; 90)	71(51; 90)	78(66; 89)	0.154
Drug therapy				
Beta-blockers, %	49	64	49	0.403
ACE inhibitors, %	31	18	32	0.448
ARA, %	17	27	16	0.536
Calcium antagonists, %	12	27	11	0.363
Statins, %	32	36	32	0.802
Nitrates, %	8	9	8	0.964
Thiazide diuretics, %	9	0	10	0.576
Loop diuretics, %	12	27	11	0.369
Potassium-sparing diuretics, %	21	36	21	0.386
Antiarrhythmic drugs, %	5	9	5	0.813

LV EF—left ventricular ejection fraction; LVEDD—left ventricular end diastolic diameter; BSA—body surface area; LVEDV—left ventricular end diastolic volume; LVESD—left ventricular end systolic diameter; LVESV—left ventricular end systolic volume; AR—aortic regurgitation, EOA—effective orifice area; LA—left atrium; WBC—white blood cells; eGFR—estimated glomerular filtration rate; MDRD—modification of diet in renal disease; ACE—angiotensin-converting enzyme; ARA—angiotensin receptor antagonist.

**Table 3:** Operational and postoperative parameters of patients.

Parameters	All	Mortality	No Mortality	*p*
N	742	22	720	
CPB time, min	137 (120; 163)	185 (149; 215)	137 (120; 161)	<0.001
ACC time, min	69 (61; 85)	78 (65; 125)	69 (61; 84)	0.029
AVR + CABG, %	13.4	22.7	13	0.440
AVR + MVR, %	7	22.7	6.5	<0.001
AVR + MV plasty,%	3.2	9	3	0.629
AVR + MV repair (in total),%	10	31.8	9.5	<0.001
AVR + TVR,%	<1	0	<1	0.982
AVR + TV plasty,%	9	22.7	8.6	<0.001
AVR + TV repair (in total),%	9.2	22.7	8.8	0.025
AVR + CryoMaze procedure,%	2.9	9	2.8	0.614
3 and more procedures,%	8.3	27.3	7.8	0.001
ICU time, days	1 (1; 1)	6 (2; 12)	1 (1; 1)	<0.001
Length of stay, days	7 (6; 9)	19 (12; 21)	7 (6; 9)	0.005

CPB—cardiopulmonary bypass; ACC—aortic cross-clamp; AVR—aortic valve replacement; CABG—coronary artery bypass grafting; MVR—mitral valve replacement; MV—mitral valve; TVR—tricuspid valve replacement; TV—tricuspid valve; ICU time – intensive care unit time.

**Table 4:** The results of ROC analysis for the initial quantitative clinical, instrumental and laboratory parameters, associated with mortality.

Parameters	Cut-Off Point	AUC (CI)	Se	Sp	*p*
Age, y	>62	0.594 (0.558–0.630)	77.3	45.6	0.594
BMI, kg/m^2^	>30	0.640 (0.605–0.675)	72.7	56.8	0.027
LVESD/BSA, cm/m^2^	>1.68	0.657(0.617–0.695)	85.0	50.80	0.009
LVEDD/BSA, cm/m^2^	>2.39	0.647 (0.607–0.686)	952	33.2	0.014
ACC time, min	>93	0.676 (0.640–0.710)	50.0	82.7	0.010
CPB time, min	>144	0.809 (0.778–0.837)	95.5	58.2	<0.001
Hemoglobin level, g/L	≤120	0.762 (0.728–0.793)	59.1	85.7	<0.001
Hematocrit, %	≤39	0.755 (0.721–0.786)	77.3	62.5	<0.001
Neutrophils, %	>68	0.657 (0.615–0.697)	50.0	87.3	0.067
Preop creatinine level, mkmoL/L	>98	0.544(0.506–0.582)	36.4	77.7	0.536
eGFR, mL/min	≤64	0.595(0.557–0.632)	40.9	83.9	0.163
eGFR, mL/min per 1,73 m^2^ (MDRD)	≤67	0.584(0.551–0.626)	50	73.5	0.212
Risk of in-hospital death, (%)	>1.4	0.722(0.687–0.754)	76.2	57.8	<0.001

AUC—area under curve; CI—confidence interval; Se—sensitivity; Sp—specificity; BMI—body mass index; BSA—body surface area; LVESD—left ventricular end systolic diameter; LVEDD—left ventricular end diastolic diameter; ACC—aortic cross-clamp; CPB—cardiopulmonary bypass; eGFR—estimated glomerular filtration rate.

**Table 5:** The results of univariate logistic regression analysis of the initial and surgical parameters.

Parameters	Univariate Logistic Regression Analysis OR (95% CI)	*p*
BMI > 30 kg/m^2^	2.84 (1.15–7.01)	0.019
LVESD/BSA cm/m^2^ > 1.68	4.81 (1.03–22.5)	0.023
LVEDD/BSA cm/m^2^ > 2.39	5.01 (1.07–23.4)	0.003
IHD	3.65 (1.01–13.2)	0.029
Prior AMI	4.16 (1.11–15.7)	0.064
Diabetes	3.88 (1.38–10.9)	0.018
Arrhythmia	2.33 (0.98–5.57)	0.063
FVE	9.78 (1.91–50.2)	0.026
AV block	5.83 (1.19–28.2)	0.067
MV repair	4.47 (1.76–11.3)	0.004
TV repair	3.06 (1.09–8.58)	0.053
3 and more procedures	4.44 (1.67–11.8)	0.007
ACC time, min > 93	4.28 (1.7–10.77)	0.003
CPB time, min > 144	25.1 (3.3–189.1)	<0.001
Hemoglobin level ≤ 120 g/L	8.67 (3.6–20.83)	<0.001
Hematocrit ≤ 39%	5.64 (2.1–15.49)	<0.001

OR – odds ratio; CI - confidence interval; BMI—body mass index; BSA—body surface area; LVESD—left ventricular end systolic diameter; LVEDD—left ventricular end diastolic diameter; IHD—ischemic heart disease; AMI—acute myocardial infarction; FVE—frequent ventricular ectopy; AV block—atrioventricular block; MV—mitral valve; TV—tricuspid valve; ACC—aortic cross-clamp; CPB—cardiopulmonary bypass.

**Table 6:** Multivariate logistic regression analysis.

Parameters	Coefficient	Standard Error	*p*
FVE	2.358	0.953	0.013
CPB time, min > 144	2.417	1.102	0.028
BMI > 30 kg/m2	1.335	0.720	0.063
IHD	1.181	0.813	0.146
Hemoglobin level ≤ 120 g/L	1.107	0.811	0.172
Hematocrit ≤ 39%	0.765	0.820	0.351
TV repair	0.703	0.806	0.383

FVE—frequent ventricular ectopy; CPB—cardiopulmonary bypass; BMI—body mass index; IHD—ischemic heart disease; TV—tricuspid valve.

## Data Availability

The datasets generated and analyzed during the current study are not publicly available due the Policies for Access to Clinical Data of Bakulev National Medical Research Center for Cardiovascular Surgery of the Ministry of Health of the Russian Federation but are available from the corresponding author upon reasonable request.
